# The Influence of Various Cereal Brans, Stabilized Through Hot Air, Microwave, and Autoclave Methods, on the Physicochemical Properties of Cookies

**DOI:** 10.1002/fsn3.70328

**Published:** 2025-05-23

**Authors:** Büşra Solmaz, Hacer Levent, Nazlı Şahin

**Affiliations:** ^1^ Faculty of Engineering, Department of Food Engineering Karamanoğlu Mehmetbey University Karaman Turkey; ^2^ Faculty of Health Sciences, Department of Nutrition and Dietetics Karamanoğlu Mehmetbey University Karaman Turkey

**Keywords:** cereal bran, cookie, physicochemical properties, stabilization

## Abstract

This study aimed to evaluate the effects of stabilizing wheat, rye, and oat brans using hot air, microwave, and autoclave methods. The stabilized brans were incorporated into cookies at 20% (w/w) based on wheat flour. The impact of bran type and stabilization method was assessed in terms of proximate composition, color, bioactive components, dietary fiber, mineral content, and texture. The results showed that both bran type and stabilization method significantly influenced the color characteristics of the cookies (*p* < 0.05). Wheat bran increased ash content, while oat bran enhanced protein, total phenolic content (TPC), and antioxidant activity (AA). Autoclaving yielded the highest TPC and AA, and the lowest phytic acid level. Cookies with wheat and rye bran had higher soluble dietary fiber (SDF) and total dietary fiber (TDF), although TDF decreased in all stabilized samples. Hot air improved insoluble dietary fiber (IDF), while microwave treatment enhanced SDF. The physical dimensions (diameter, thickness, spread ratio) and textural properties (hardness, fracturability) of cookies were only slightly affected. Wheat bran increased calcium, magnesium, and zinc levels by 1.5‐, 1.9‐, and 1.9‐fold, respectively. Microwave and autoclave treatments further elevated potassium, concentrations. These findings suggest that stabilized cereal brans can be effectively utilized to formulate fiber‐ and mineral‐rich cookies with improved antioxidant capacity. Microwave and autoclave treatments, in particular, show promise for developing functional bakery products targeted at health‐conscious consumers. The application of these techniques also aligns with sustainable and value‐added food production strategies, supporting broader industrial use.

## Graphical abstract

Stabilization methods, including hot air, microwave, and autoclave treatments, can significantly enhance the nutritional value of bran derived from wheat, rye, and oats, all while preserving the quality of cookies. By implementing these techniques, food manufacturers have the opportunity to develop cookies that are not only fiber‐rich and nutritious but also functional, effectively addressing the nutritional deficiencies linked to the consumption of refined foods. Moreover, the stabilization of cereal by‐products supports sustainable food processing practices.

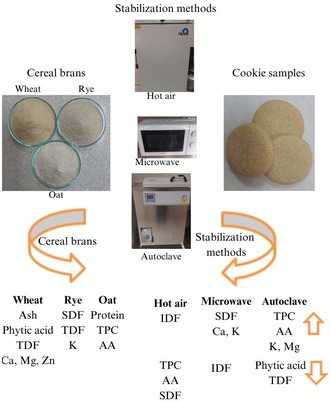

## 1 Introduction

The upward trend in unhealthy eating habits, the consumption of energy‐dense foods, and the parallel sedentary lifestyle have contributed significantly to the alarming increase in health risks such as obesity and diabetes, which have now reached epidemic proportions (Blüher and Stumvoll 2020). The focus on the link between chronic diseases and nutrition in numerous scientific studies has heightened awareness of healthy eating (Anand et al. 2020).

Cookies are a significant snack product due to their long shelf life and the variety of flavors they offer consumers. The high sugar and fat content and low fiber levels contribute to unhealthy nutrition, discouraging consumption. Therefore, extensive research is being conducted to reduce the sugar and fat content while increasing dietary fiber and bioactive component levels (Anand et al. 2020; Goswami et al. 2021; Kumar et al. 2023).

Cereal brans, byproducts of cereal processing, are rich in health‐promoting compounds. Brans from cereals like rice, wheat, oats, and barley contain dietary fiber, vitamins (especially B and E), and biologically active compounds including alkylresorcinol, ferulic acid, β‐glucan, and arabinoxylan (Fărcaș et al. 2021). Cereal bran is also an important source of phenolic acids, including derivatives of hydroxybenzoic acid and hydroxycinnamic acid, which are recognized as important contributors to the reported protective effect of whole grain consumption against chronic diseases (Martín‐Diana et al. 2021).

Wheat bran contains protein (13%–18%), fat (3%–5%), and carbohydrates (50%–60%), of which 70%–90% is dietary fiber (Kong et al. 2021; Jimenez‐Pulido et al. 2022). Due to its high levels of non‐starch polysaccharides (especially arabinoxylans and β‐glucans), wheat bran has health benefits in the treatment and prevention of obesity, cardiovascular disease, cholesterol, type 2 diabetes, and cancer (Sztupecki et al. 2023). Rye bran is rich in arabinoxylan, cellulose, β‐glucan, lignin, and fructan, which help maintain a healthy body weight and reduce the incidence of diabetes, heart disease, and cancer (El‐Mahis et al. 2023). Oat bran is rich in phenolic compounds with high antioxidant activity and has a higher soluble fiber content than wheat or rice bran. This is important in the prevention of obesity, diabetes, and cardiovascular disease (Saka et al. 2021).

Global nutritional guidelines recommend increasing the intake of foods rich in cereal bran and whole grains (Mathews and Chu 2020). Despite its abundance and richness in fiber, phytochemicals, micronutrients, and bioactive compounds, it is under‐utilized for human consumption and is mostly used as animal feed due to ignorance, spoilage problems, and organoleptic reasons (Ashraf et al. 2022; Sztupecki et al. 2023). The most important factor causing spoilage and sensory problems in cereal bran is the enzymes present in the embryo and bran of the grain. Of these enzymes, lipase plays a major role in causing rancidity by hydrolyzing triacylglycerols to fatty acids. Lipoxygenase is also present in bran and facilitates the enzymatic oxidation of free fatty acids (Bhunia et al. 2023).

Various treatments, including microwave heating, autoclaving, infrared heating, steaming, and roasting, have been documented in the literature to effectively stabilize and extend the shelf life of wheat bran and wheat germ (Demir and Elgün 2014; Demir et al. 2019). Both microwave and autoclave treatments have been shown to significantly increase the total phenolic content and antioxidant activity, primarily by disrupting cell walls and vacuoles, which facilitates the release of bound phenolic acids (Saroj et al. 2024). This enhancement is also attributed to thermal degradation reactions such as the Maillard reaction and the hydrolysis of ester linkages between phenolic acids and polysaccharides (Ragaee et al. 2014; Saroj et al. 2024). Stabilization treatments effectively reduced the antinutritional components in cereal brans. Iraklı et al. (2020) investigated stabilization treatments for rice bran using microwave, infrared radiation, and dry heating methods. Their findings indicated significant reductions in antinutritional components such as phytate, oxalate, saponins, and trypsin inhibitors. The microwave treatment achieved the highest reduction in phytate content, approximately 26%, followed by infrared radiation at around 22.2% and dry heating at about 19.9%. Stabilization also enhances total and HCl‐extractable mineral content in whole wheat bread, particularly with autoclave and microwave methods (Demir and Elgün 2014).

To the best of our knowledge, there are no studies on using stabilized different cereal brans in cookie production. Therefore, the aim of this research is (1) to evaluate the effects of wheat, rye, and oat bran stabilized with different methods (hot air, microwave, and autoclave) on the physicochemical quality of cookies and (2) to compare the effects of cereal bran type and stabilization methods on cookie quality.

## 2 Materials and Methods

### 2.1 Materials

Wheat flour, powdered sugar, shortening, salt, baking powder, and ethyl vanillin used in cookie production were purchased from local markets in Karaman, Türkiye. Skimmed milk powder was sourced from Ova Dairy Products Inc. in Konya, Türkiye. Wheat bran was obtained from a flour mill, while oat and rye bran were purchased from Health Agricultural Products and Food Industry, also located in Konya, Türkiye.

### 2.2 Methods

#### 2.2.1 Stabilization of Cereal Brans

The stabilization process was conducted according to the method outlined by Demir et al. (2019). Before this process, all bran was passed through a 500 μm mesh sieve. The stabilization standards applied to cereal brans are as follows:

The hot air stabilization process involved monitoring the internal temperature of 100 g bran samples until they reached 100°C with an IR thermometer (Ebro TLC 730, Germany). Stabilization was maintained at this temperature for 1 min. The oven (Nüve KD‐200, Ankara, Turkey) was set to 160°C, requiring 23 to 25 min to meet stabilization standards.

For stabilization with a microwave, cereal bran (100 g) was placed evenly in a glass container with a depth of 3 mm and heated in a microwave oven (Arçelik, 2450 MHz, 700 W). The stabilization process involved maintaining the lid closed for an additional minute after the internal temperature of the bran reached 70°C, without further heating. The total time required to achieve stabilization standards in the microwave was approximately 5 minutes.

For stabilization with an autoclave, 100 g of cereal bran was securely placed in autoclavable bags, which were tightly sealed to prevent moisture ingress. The samples were then subjected to autoclaving (Nüve, Steam Art OT) at 121°C for 15 min.

#### 2.2.2 Cookie Production

Cookies were prepared using the modified AACC (10.54) method (AACC 2010). The control sample included wheat flour (100 g), powdered sugar (40 g), shortening (40 g), salt (1 g), skimmed milk powder (1 g), baking powder (2 g), ethyl vanillin (0.5 g), and water (15 mL). These ingredients were mixed in the mixer (Kitchen Aid Series, USA) at low speed (Speed 1) for 5 min until homogeneous. The dough was rolled to 5 mm thickness, cut into 50 mm circles, and baked at 160°C for 18 min in an oven (Arçelik 8431, Türkiye). In enriched cookie samples, wheat flour was replaced by 20% of the wheat flour with wheat, rye, and oat bran. A control cookie without cereal bran and a cookie with untreated cereal bran were produced for comparison. After baking, the cookies were cooled at room temperature and stored in polyethylene bags until analysis.

#### 2.2.3 Physical Properties of the Cookie Samples

The color values (L*, a*, and b*) of the cookie samples were measured using a Chroma‐meter (Konica Minolta, Japan). The saturation index (SI) was calculated using SI = (a*^2^ + b*^2^)^1^/^2^. The hue angle was determined using the following formulas: Hue = Arctan (b*/a*) + 180 when *a* < 0 and *b* > 0, and Hue = Arctan (b*/a*) when *a* > 0 and *b* > 0, following the method outlined by Francis (1998).

The diameter and thickness of the cookies were measured with a caliper (Mitutoyo, Tokyo, Japan). The spread ratio was then calculated by dividing the cookie diameter by thickness.

The hardness and fracturability of the cookies were measured using a TA‐XT2i texture analyzer (Stable Micro Systems Ltd., UK) with a three‐point bending ring (HDP/3 PB). The measurement parameters were set as follows: pre‐test speed at 1.00 mm/s, test speed at 3.00 mm/s, post‐test speed at 10.00 mm/s, trigger force at 50.0 g, and a distance of 5 mm. The hardness and fracturability values were recorded 2 h after baking.

#### 2.2.4 Chemical Analysis of the Cookie Samples

AACC standard methods were used for moisture (44–19), ash (08–01), protein (46–12), and fat (30–25). Phytic acid content was measured using the colorimetric method by Haug and Lantzsch (1983). A 0.3 g sample of wheat flour, cereal brans, or cookies was extracted with 0.2 N hydrochloric acid. Then, 0.5 mL of the extract was mixed with 1 mL of ammonium iron (III) sulfate, heated for 30 min, and cooled in an ice bath for 15 min. Finally, 2 mL of 2,2′‐bipyridine was added, and absorbance was measured at 519 nm using a Shimadzu spectrophotometer (UV1800, Japan).

Total phenolic content (TPC) was measured using the Folin–Ciocalteu method (Beta et al. 2005). A 1 g sample was extracted with 10 mL of acidified methanol (methanol: distilled water: HCl, 80:10:1, v/v/v) for 2 h at room temperature. The extract (0.1 mL) was mixed with 1.5 mL sodium carbonate (20%, w/v), 0.5 mL of Folin‐Ciocalteu reagent (10% diluted, v/v), and 7.9 mL of distilled water in the tube then incubated in the dark for 2.5 h. After incubation, the absorbance value was measured using a spectrophotometer (Shimadzu UV1800, Japan) at 760 nm, and the TPC was expresses as mg gallic acid equivalents/kg.

The antioxidant activities of the samples (AA) were assayed according to the 2,2‐diphenyl‐2‐picrylhydrazyl (DPPH) method. The samples were extracted as indicated for TPC analysis. Absorbance values were read in a spectrophotometer at 517 nm, and AA was determined as percent inhibition (Gyamfi et al. 1999; Beta et al. 2005).

The analysis of dietary fiber in the cereal brans and cookie samples was performed using the AOAC 960.43 method and the Megazyme Dietary Fiber Determination Kit (K‐TDFR‐100A, Ireland). This technique enabled the quantification of insoluble dietary fiber (IDF) and total dietary fiber (TDF). To determine the soluble dietary fiber (SDF), the IDF was subtracted from the TDF, as outlined by AOAC (2005).

For mineral analysis, 1 g of the sample was mixed with 15 mL of nitric acid overnight. Then, 4 mL of perchloric acid was added and gently heated for 5–6 h. After cooling, 5 mL of hydrogen peroxide was added, and the samples were reheated until discoloration occurred. Mineral concentration was determined using ICP OES (Agilent 720, USA).

### 2.3 Statistical Analysis

The results are expressed as means ± standard deviations. Data was analyzed using SPSS software (Version 22.0, Armonk, NY: IBM Corp.). Variance analysis was performed, and the averages of the primary sources of variation were compared utilizing the Duncan multiple comparison test. The values of *p* < 0.05 were deemed statistically significant. The data represent the mean of triplicate measurements obtained from duplicate experiments.

## 3 Results and Discussion

### 3.1 Chemical Properties of Raw Materials

The color values and chemical compositions of wheat flour, wheat bran, rye bran, and oat bran are shown in Table 1. As expected, wheat flour had the highest L* value (93.23), Hue (92.90), and the lowest a* (−0.50), b* (10.02), and SI (10.03) values among the raw materials. Lower L* and higher a* and b* values were determined in all cereal brans due to the concentration of color pigments in the outer layers of the grain. The moisture, ash, protein, and fat values of raw materials ranged between 6.33%–9.89%, 0.62%–3.87%, 9.56%–16.52%, and 0.87%–3.31%, respectively. Compared to other brans, the ash content was higher in wheat bran, whereas the protein content was higher in rye and oat bran than in wheat bran. Martín‐Diana et al. (2021) reported similar findings, noting that wheat bran had an ash content of 7.31% and a protein content of 13.19%. In comparison, oat bran displayed a lower ash content of 3.03% but a significantly higher protein content of 17.63%. These results suggest that oat bran is a more advantageous protein source, while wheat bran is characterized by its elevated ash content. In comparison to wheat flour, cereal bran exhibits significantly higher nutritional values, demonstrating 4.8 to 6.2 times more ash content, 1.5 to 1.7 times more protein, and 2.8 to 3.8 times more fat content.

**TABLE 1:** Chemical properties and color values of raw materials.

	Wheat flour	Wheat bran	Rye bran	Oat bran
L*	93.23 ± 0.27a	77.58 ± 0.33c	72.90 ± 0.21d	84.71 ± 0.35b
a*	−0.50 ± 0.13d	3.39 ± 0.07a	2.87 ± 0.10b	1.38 ± 0.08c
b*	10.02 ± 0.30c	20.12 ± 0.18a	14.62 ± 0.14b	13.95 ± 0.20b
SI	10.03 ± 0.22c	20.40 ± 0.40a	14.90 ± 0.25b	14.02 ± 0.20b
Hue	92.90 ± 0.21a	80.44 ± 0.28c	78.89 ± 0.32d	84.35 ± 0.18b
Moisture (%)	9.89 ± 0.16a	6.33 ± 0.22b	8.29 ± 0.31b	7.79 ± 0.19b
Ash (%)	0.62 ± 0.06c	3.87 ± 0.10a	3.10 ± 0.08b	2.97 ± 0.13b
Protein	9.56 ± 0.16c	14.03 ± 0.25b	16.18 ± 0.20a	16.52 ± 0.28a
Fat (%)	0.87 ± 0.16c	3.09 ± 0.21ab	2.44 ± 0.18b	3.31 ± 0.23a
SDF (%)	2.24 ± 0.06d	4.77 ± 0.13b	3.67 ± 0.05c	5.14 ± 0.00a
IDF (%)	1.32 ± 0.00c	48.57 ± 0.34a	22.53 ± 0.82b	23.81 ± 1.00b
TDF (%)	3.55 ± 0.06c	53.34 ± 0.22a	26.20 ± 0.77b	28.49 ± 1.67b
Phytic acid (mg/100 g)	188.30 ± 6.28d	2631.99 ± 8.22a	2325.70 ± 5.03b	2049.99 ± 7.52c
TPC (mg GAE/kg)	465.40 ± 7.47d	1713.09 ± 9.22b	1666.92 ± 7.81c	2105.38 ± 5.95a
AA (%)	10.40 ± 0.21d	36.52 ± 0.16b	35.68 ± 0.25c	42.36 ± 0.17a
Mineral content (mg/100 g)				
Ca	30.84 ± 0.68d	97.83 ± 0.40a	57.31 ± 0.51c	72.55 ± 0.42b
Fe	1.36 ± 0.13c	7.12 ± 0.28a	6.58 ± 0.17ab	6.12 ± 0.33b
K	138.40 ± 1.22d	785.66 ± 1.57b	820.52 ± 2.26a	506.70 ± 1.15c
Mg	34.50 ± 1.44d	236.87 ± 1.03a	210.65 ± 2.42c	227.58 ± 1.60b
Zn	1.16 ± 0.16d	6.04 ± 0.20a	3.31 ± 0.14b	2.83 ± 0.11c

*Note:* Chemical properties, except moisture, are based on dry matter. The values followed by different lowercase letters within a row are significantly different (*p* ≤ 0.05).

Abbreviations: AA, Antioxidant activity; GAE, Gallic acid equivalent; Hue, Hue value; IDF, Insoluble dietary fiber; SDF, Soluble dietary fiber; SI, Saturation index; TDF, Total dietary fiber; TPC, Total phenolic content.

The chemical components of cereals are distributed unevenly among the various anatomical parts of the grains. The bran, serving as the protective envelope of the grain, is primarily composed of proteins, fats, dietary fiber, vitamins, minerals, and phytochemicals (Hadidi et al. 2024).

Dietary fiber can be categorized into soluble dietary fiber (SDF), insoluble dietary fiber (IDF), and total dietary fiber (TDF). Cereal brans generally contain significantly higher dietary fiber levels than wheat flour. The SDF, IDF, and TDF amounts (%) in wheat flour are 2.24, 1.32, and 3.55, respectively. In contrast, the SDF values of cereal brans range from 3.67% to 5.14%, IDF values range from 22.53% to 48.57%, and TDF values range from 26.20 to 53.34. Additionally, wheat bran has IDF and TDF values twice as high as rye and oat bran. Cereal brans are an important source of dietary fiber, which aids in maintaining normal gastrointestinal function. They help to reduce glucose and fat absorption, slow the hydrolysis of starches, and provide probiotic benefits for intestinal bacteria. These brans contribute to an increase in intestinal volume, shorten food transit time, and lower the risk of constipation, obesity, diabetes, appendicitis, and intestinal and colon cancers (Stephen et al. 2017).

The phytic acid content of the raw materials analyzed ranged from 188.30 to 2631.99 mg/100 g. Notably, wheat bran exhibited the highest phytic acid concentration in this study. Among cereal brans, the highest TPC and AA values were determined in oat bran, followed by wheat and rye bran, respectively. Phytochemical compounds found especially in the bran part of grains possess antioxidant activity (Oghbaei and Prakash 2016). Leszczyńska et al. (2023) highlighted that whole oat groats are abundant in essential nutrients, such as proteins, soluble fibers, unsaturated fatty acids, phytochemicals, vitamins, and minerals. Furthermore, a study by Levent et al. (2020) revealed that the phytic acid contents in wheat, rye, and oat bran were 2573.18, 2164.38, and 2030.79 mg/100 g, respectively.

Ca, K, Mg, Fe, and Zn values in raw materials ranged between 30.84–97.83, 138.40–820.52, 34.50–236.87, 1.36–7.12, and 1.16–6.04 mg/100 g, respectively (Table 1). While Ca, Fe, Mg, and Zn were found to be highest in wheat bran, K was found to be highest in rye bran. Wheat bran had higher Fe content than oat bran. Wheat bran was reported to have higher Ca, P, Fe, Mg, and Zn content than oat bran (Levent et al. 2020). Minerals are predominantly located in the outer bran and aleurone layers of cereal grains. Consequently, the nutritional quality of cereal flour is significantly influenced by the extent to which these layers are removed during processing (Oghbaei and Prakash 2016).

### 3.2 Physical and Textural Properties of Cookie Samples

**FIGURE 1:**
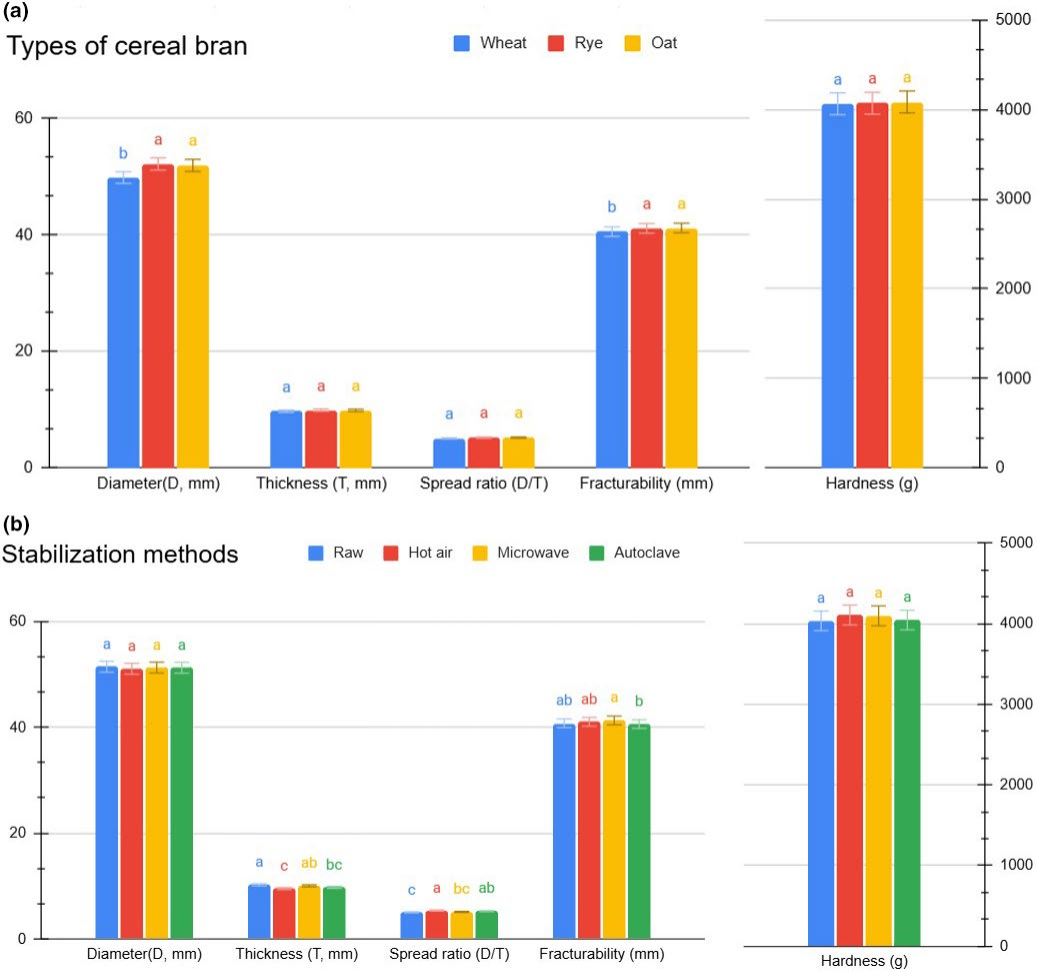
The effects of cereal types (a) and stabilization methods (b) of the diameter, thickness, spread ratio, fracturability, and hardness of cookies. Different letters indicate statistically significant differences among the values (*p* < 0.05).

The color values of cookies containing cereal bran are given in Table 2. When the color values were evaluated according to the type of cereal bran, the highest L*, b*, SI, and Hue values were determined in the cookie samples containing oat bran, followed by the cookies containing wheat bran and rye bran, respectively. It has been reported that the natural color of the bran, as well as the higher amount of protein in biscuits containing bran, may increase the Maillard reaction (Sözer et al. 2014). When the color analysis results were evaluated according to the stabilization method, it was determined that the lowest L* value in cookies was obtained in the autoclave and then in the microwave method. Higher a* values were obtained in these two methods compared to other methods. In addition, lower a*, b*, and SI values were obtained in the hot air stabilization method compared to other stabilization processes. The lower L* value in cookie samples containing cereal bran stabilized by microwave and autoclave methods can be attributed to the fact that the temperature and duration to which the cereal bran is exposed in these methods may affect the Maillard reaction. In the study by Demir and Elgün (2013), two different wheat samples were ground, and the resulting branny fractions were stabilized using microwave, autoclave, ultraviolet, and infrared methods. The processed “whole wheat fractions” were then reblended with the separated white flour from the same samples to produce whole wheat flour. The most notable effects were observed with the autoclave and microwave treatments, attributed to their superior heat penetration properties. This impact was thought to be related to Maillard and caramelization reactions (Alkanan et al. 2024). The thickness, spread ratio and hardness, values of cookie samples were not found to be significantly different from each other according to the cereal bran type (Figure 1a). The effects of the stabilization method on the thickness, spread ratio, and fracturability properties of cookies were statistically different (*p* < 0.05), but the results were measured to be quite close to each other (Figure 1b).

**TABLE 2:** Multiple comparison test results of color values of cookie samples supplemented with cereal brans stabilized by different methods.

	*n*	L*	a*	b*	SI	Hue
Type of cereal bran						
Wheat	8	68.67 ± 1.83b	4.16 ± 0.32a	26.81 ± 0.69b	27.13 ± 0.68b	81.18 ± 0.70b
Rye	8	66.53 ± 0.96c	4.28 ± 0.60a	25.99 ± 1.37c	26.34 ± 1.44c	80.70 ± 0.86c
Oat	8	70.83 ± 1.05a	3.35 ± 0.55b	27.75 ± 0.79a	27.95 ± 0.84a	83.13 ± 0.91a
Stabilization method						
Raw	6	69.54 ± 1.42a	3.86 ± 0.63b	27.12 ± 0.55ab	27.40 ± 0.48b	81.89 ± 1.40b
Hot air	6	69.36 ± 3.23a	3.39 ± 0.32c	25.83 ± 1.57c	26.05 ± 1.55c	82.49 ± 0.95a
Microvawe	6	68.24 ± 1.45b	4.29 ± 0.11a	27.59 ± 1.16a	27.93 ± 1.14a	81.14 ± 0.48c
Autoclave	6	67.56 ± 2.14c	4.17 ± 0.88a	26.85 ± 0.72b	27.18 ± 0.67b	81.16 ± 1.90c
Control		73.65 ± 0.35	1.82 ± 0.11	28.39 ± 0.27	28.45 ± 0.23	86.33 ± 0.30

*Note:* The values followed by different lowercase letters within a column are significantly different (*p* < 0.05).

Abbreviations: a*, Red–green color value; b*, Yellow–blue color value; Hue, Hue value; L*, Lightness value; SI, Saturation index.

### 3.3 Chemical Properties of Cookies

The results of the chemical analyses for the cookie samples are detailed in Table 3. Based on the multiple comparison tests conducted according to the cereal bran type, the moisture content of cookie samples ranged from 3.52% to 4.49%. The cookie samples made with wheat bran exhibited a higher ash content than those containing rye or oat bran. Specifically, the raw materials analysis revealed that wheat bran had a higher ash content (3.87%) than rye bran (3.10%) and oat bran (2.97%, refer to Table 1). This differential in raw material composition is likely reflected in the final product. Additionally, it was observed that cookies made with oat bran contained a higher protein content compared to those made with wheat bran. In the study conducted by Sözer et al. (2014), 2 different sizes of wheat bran were used in different ratios (5%–15%) in the production of biscuits. It has been reported that samples containing higher amounts of wheat bran contain higher amounts of protein; while the amount of protein in the control sample was 6.84%, it increased to 8.63% in samples containing 30% wheat bran. According to the stabilization method, the ash, protein, and fat contents of the cookie samples were not significantly different from each other. Compared to the control, the use of cereal bran caused an increase in the amount of ash and protein in cookies. Makowska et al. (2015) explored the incorporation of oat, wheat, and rye bran at levels of 20% and 40% into extruded corn snacks. Their findings indicated that snacks enriched with oat bran exhibited the highest protein and fat content, whereas the mineral content was comparatively lower.

**TABLE 3:** Multiple comparison test results of chemical properties of cookie samples supplemented with cereal brans stabilized by different methods.

	*n*	Moisture (%)	Ash (%)	Protein (%)	Fat (%)	Phytic acid (mg/100 g)	TPC (mg GAE/kg)	AA (% inhibition)	SDF (%)	IDF (%)	TDF (%)
Type of cereal bran											
Wheat	8	4.49 ± 0.51a	1.86 ± 0.08a	8.36 ± 0.15b	16.39 ± 0.25a	586.65 ± 39.30a	1190.00 ± 94.63b	16.49 ± 5.24b	7.85 ± 1.25ab	7.22 ± 0.51a	15.27 ± 1.20a
Rye	8	3.52 ± 0.21c	1.70 ± 0.07b	8.42 ± 0.16ab	16.65 ± 0.22a	502.62 ± 52.26b	1110.40 ± 91.04c	14.44 ± 4.28c	9.55 ± 2.15a	5.80 ± 2.07a	15.35 ± 1.00a
Oat	8	4.07 ± 0.37b	1.67 ± 0.06b	8.65 ± 0.16a	16.26 ± 0.21a	460.89 ± 39.64c	1237.47 ± 58.44a	20.79 ± 6.41a	5.85 ± 3.05b	6.77 ± 2.45a	12.63 ± 2.88b
Stabilization method											
Raw	6	4.16 ± 0.54a	1.75 ± 0.13a	8.40 ± 0.20a	16.43 ± 0.28a	571.44 ± 56.07a	1141.25 ± 73.59c	13.14 ± 1.94c	9.19 ± 1.74ab	6.49 ± 1.31b	15.68 ± 1.49a
Hot air	6	4.19 ± 0.58a	1.73 ± 0.10a	8.47 ± 0.17a	16.42 ± 0.29a	532.40 ± 56.23b	1075.62 ± 71.05d	11.88 ± 1.80d	6.00 ± 2.40c	8.68 ± 1.29a	14.67 ± 1.66ab
Microwave	6	3.74 ± 0.29b	1.74 ± 0.12a	8.52 ± 0.24a	16.44 ± 0.30a	502.30 ± 60.22c	1232.43 ± 45.23b	20.87 ± 5.60b	9.33 ± 2.43a	4.94 ± 1.65b	14.52 ± 2.29ab
Autoclave	6	4.01 ± 0.71ab	1.75 ± 0.11a	8.52 ± 0.20a	16.44 ± 0.29a	460.73 ± 59.03d	1267.86 ± 46.56a	23.08 ± 2.35a	6.50 ± 2.63bc	6.29 ± 1.34b	12.78 ± 2.70b
Control		5.11 ± 0.13	1.38 ± 0.11	7.61 ± 0.14	16.10 ± 0.33	197.20 ± 6.22	862.45 ± 6.15	10.58 ± 0.25	7.57 ± 0.43	1.87 ± 0.43	9.43 ± 0.00

*Note:* The values followed by different lowercase letters within a column are significantly different (*p* < 0.05). Results except moisture are based on dry matter.

Abbreviations: AA, Antioxidant activity; IDF, Insoluble dietary fiber; SDF, Soluble dietary fiber; TDF, Total dietary fiber; TPC, Total phenolic content.

According to the cereal bran type, the highest amount of phytic acid was determined in cookies containing wheat bran (586.65 mg/100 g), followed by samples containing rye (502.62 mg/100 g) and oat bran (460.89 mg/100 g), respectively. In the raw material analysis results, the high phytic acid content of wheat bran was found remarkable. Among the cereal brans used, the lowest phytic acid content was determined in oat bran (Table 1). Subtain et al. (2024) studied the effects of different fermentation processes on the composition of cereal brans (wheat, sorghum, barley, oats, and maize). It was reported that the highest phytic acid content in cereal brans (in its native form) was found in wheat bran (180.59 mg/100 g) and the lowest in oat bran (147.10 mg/100 g).

According to the stabilization method, the lowest amount of phytic acid in cookie samples was determined in the autoclave method and then in the microwave and hot air methods, respectively. The highest amount of phytic acid was determined in cookies containing raw (untreated) cereal bran (Table 3). In the study conducted by Ertaş (2015), wheat bran (WB) stabilized with a microwave oven, hot air oven, and the autoclave was used in the production of cookies at three different ratios (10%, 20%, and 30%). The lowest phytic acid content was obtained in cookie samples containing oat bran stabilized by autoclave among various stabilization methods. This result is consistent with previous findings reporting that autoclaving oat bran (pH 4.0 for 1.5 h) led to a 95.2% reduction in phytic acid content, which was higher than reductions achieved by fermentation or autoclaving without pH adjustment (Özkaya et al. 2017).

When multiple comparison test results were examined according to cereal bran type, TPC and AA values of cookie samples containing wheat bran, rye bran, and oat bran were determined as 1190.00, 1110.40, and 1237.47 mg GAE/kg (TPC), 16.49%, 14.44%, and 20.79% (AA), respectively. The highest TPC and AA values were determined in cookie samples containing oat bran, followed by samples containing wheat and rye bran, respectively. Oats ( *Avena sativa* L.) are a valuable source of numerous compounds recognized for their antioxidant properties. Among the most prevalent antioxidants present in oats are vitamin E (tocopherols), phenolic compounds, phytic acid, and avenanthramides. Additionally, oats contain sterols and flavonoids, contributing to their overall antioxidant profile (Li et al. 2024).

Phenolic compounds in cereals are primarily located in the outer layer rather than in the endosperm. Literature has reported a strong positive correlation between total phenolic compounds and the antioxidant activity of phenolic extracts (Van Hung 2016). Bioactive phenolic compounds in cereal grains are predominantly located in the bran fraction, where they are covalently bonded to indigestible polysaccharides (Wang et al. 2014). Microwave and autoclave methods caused an increase in TPC in cookie samples. This increase due to thermal treatment may be due to the release of bound phenolic acids due to the degradation of cellular components and cell walls. In addition, browning during thermal treatment results in an increase in total phenolic content and free radical scavenging capacity. The observed increase in phenolic content may result from the decomposition of conjugated phenolic compounds during thermal treatment, which leads to subsequent polymerization and oxidation reactions. This process can also generate phenolics not originally found in the grains. Additionally, other reactions, such as the Maillard reaction, caramelization, and the chemical oxidation of phenols, may contribute to the overall increase in total phenolic content (Demir and Elgün 2014; Ragaee et al. 2014). Furthermore, thermal treatment hydrolyzes the ester linkages between phenolic acids and polysaccharides, increasing the concentration of phenolic acids in wheat bran after stabilization (Rico et al. 2020).

According to the stabilization methods, the highest total phenolic content (TPC) and antioxidant activity (AA) in cookies were found in samples containing bran that was stabilized using an autoclave. This result can be attributed to the high pressure and temperature during autoclaving, followed by rapid depressurization. This combination disrupts the cell wall structure and partially hydrolyzes fiber polysaccharides, which facilitates the release of bound phenolic compounds (Wang et al. 2014). In a study conducted by Saroj et al. (2024), bran from six different wheat cultivars was subjected to various heat treatments, including autoclaving, toasting, hot air drying, and microwave heating, to evaluate the effects on bioactive composition. The findings indicated that heat treatment significantly increased ferulic acid, gallic acid, syringic acid, p‐hydroxybenzoic acid, vanillic acid, and p‐coumaric acid levels. The increase in antioxidant activity can be attributed to the heat treatments, which not only augment the availability of phenolic compounds with antioxidant properties but also enhance the extractability of other bioactive antioxidants or lead to the formation of products with strong antioxidant activity, such as those produced through the Maillard reaction (Demir and Elgün 2014; Wang et al. 2014).

Based on the type of cereal bran used, cookies made with rye and wheat bran exhibited the highest total dietary fiber (TDF) content at 15.35% and 15.27%, respectively. Conversely, cookies containing oat bran had the lowest TDF, noting 12.63%. The soluble dietary fiber (SDF) levels were 9.55% for rye bran, 7.85% for wheat bran, and 5.85% for oat bran. Furthermore, no significant differences were observed in the insoluble dietary fiber (IDF) amounts. Adults should aim for 25 to 35 g of fiber for the best health benefits. This guideline differs by gender: women are recommended to intake between 25 and 32 g daily, whereas men should strive for 30 to 35 g daily (Stephen et al. 2017). Enriched cookie samples can be a good source of dietary fiber.

According to the stabilization method, the TDF amount decreased across all approaches. A reduction in total dietary fiber was observed through the application of thermal treatments. This phenomenon can be attributed to the breaking of glycosidic linkages, which resulted in hydrolysis and consequently led to the loss of arabinoxylans (Saroj et al. 2024). In contrast, the IDF amount increased with the hot air method. No significant differences were observed in IDF amount for the raw, microwave, and autoclave methods (*p* > 0.05). However, microwave treatment notably increased the SDF amount compared to the other method. The lowest levels were seen with the hot air and autoclave methods regarding SDF amounts. Saroj et al. (2024) found that microwave treatment of wheat bran led to an increase in SDF and a decrease in both IDF and TDF compared to untreated wheat bran. In this study, cookies with microwave‐treated cereal brans contained the highest levels of SDF, alongside lower amounts of IDF and TDF.

Mineral content analysis according to cereal bran type indicated that wheat bran‐enriched cookies contained significantly higher amounts of calcium (Ca), magnesium (Mg), and iron (Fe), while rye bran‐based cookies were characterized by higher potassium (K) levels (Table 4). According to the stabilization method, higher Ca, K and Mg values were obtained in cookie samples in the microwave and autoclave methods. In a study by Demir and Elgün (2014), wheat bran fractions were stabilized using microwave, autoclave, ultraviolet, and infrared methods and then remixed with white flour to produce whole wheat flour. The research showed that stabilization increased the total mineral content and HCl‐extractable minerals in whole wheat bread, with the autoclave and microwave methods yielding the most significant improvements due to better heat penetration.

**TABLE 4:** Multiple comparison test results of mineral contents of cookie samples supplemented with cereal brans stabilized by different methods.

	*n*	Ca (mg/100 g)^2^	Fe (mg/100 g)^2^	K (mg/100 g)^2^	Mg (mg/100 g)^2^	Zn (mg/100 g)^2^
Type of cereal bran						
Wheat	8	42.36 ± 3.26a	2.23 ± 0.15a	231.90 ± 3.61b	52.15 ± 2.63a	1.83 ± 0.11a
Rye	8	33.55 ± 1.73 ± c	2.14 ± 0.10a	250.35 ± 4.19a	43.02 ± 2.53c	1.12 ± 0.17b
Oat	8	36.18 ± 1.66b	2.03 ± 0.11a	204.78 ± 5.58c	48.12 ± 3.78b	0.98 ± 0.16b
Stabilization method						
Raw	6	35.17 ± 3.26c	2.09 ± 0.16a	226.06 ± 22.08b	45.48 ± 5.09c	1.28 ± 0.42a
Hot air	6	35.70 ± 3.48c	2.10 ± 0.16a	225.13 ± 19.98b	45.47 ± 3.46c	1.26 ± 0.45a
Microwave	6	40.18 ± 4.99a	2.16 ± 0.15a	233.71 ± 21.18a	48.73 ± 3.97b	1.37 ± 0.44a
Autoclave	6	38.38 ± 4.57b	2.18 ± 0.11a	231.14 ± 18.33a	51.39 ± 4.75a	1.32 ± 0.42a
Control		28.52 ± 0.59	1.28 ± 0.10	130.50 ± 2.76	27.82 ± 0.95	0.94 ± 0.13

*Note:* The values followed by different lowercase letters within a column are significantly different (*p* < 0.05). Results are based on dry matter.

## 4 Conclusion

This study explored how different stabilization methods—hot air, microwave, and autoclave—affect the technological and nutritional properties of cookies enriched with wheat, rye, and oat bran. The results indicated that the choice of stabilization method and the type of bran had a significant impact on the color, dietary fiber composition, and mineral content of the cookies. However, their physical and textural properties remained largely unchanged. Notably, the microwave stabilization method improved the levels of soluble dietary fiber and enhanced mineral content. In contrast, the thermal methods resulted in variations in total and insoluble dietary fiber levels.

These findings underscore the potential of stabilization techniques to enhance the functional and nutritional characteristics of bran‐enriched baked goods. This research offers valuable insights for food manufacturers aiming to create cookies that are rich in fiber and antioxidants. However, further studies are necessary to investigate the effects of these methods on large‐scale production, consumer acceptance, and long‐term storage stability. Additionally, examining the structural changes in bran components resulting from stabilization could provide a deeper understanding of their functional roles in bakery formulations.

## Author Contributions

**Büşra Solmaz:** data curation (lead), formal analysis (lead). **Hacer Levent:** data curation (supporting), formal analysis (supporting), writing – review and editing (equal). **Nazlı Şahin:** data curation (supporting), formal analysis (supporting), writing – review and editing (equal).

## Conflicts of Interest

The authors declare no conflicts of interest.

## Data Availability

The data that support the findings of this study are available from the corresponding author upon reasonable request.

## References

[fsn370328-bib-0001] AACC . 2010. Approved Methods of the AACC. Cereals and Grains Association.

[fsn370328-bib-0002] Alkanan, Z. T. , A. B. Altemimi , N. A. Hassan , et al. 2024. “Effects of Microwave Utilization on the Color Properties of Food: A Review.” ChemBioEng Reviews 11, no. 3: 483–494. 10.1002/cben.202300067.

[fsn370328-bib-0003] Anand, J. , G. Kandwal , M. Nath , et al. 2020. “Green Tea Enhances Nutritional and Antioxidant Potential of Pearl Millet Based Cookies: A Healthy Approach.” International Journal of Current Research and Review 12, no. 18: 48–54. 10.31782/IJCRR.2020.121812.

[fsn370328-bib-0004] AOAC . 2005. Approved Methods of the Association of Official Agricultural Chemists. United States Department of Agriculture.

[fsn370328-bib-0005] Ashraf, S. , M. Sood , J. D. Bandral , N. Gupta , and R. Rahman . 2022. “Effect of Different Stabilization Methods on Proximate and Mineral Composition of Wheat Bran.” Indian Journal of Ecology 49, no. 5: 2033–2041. 10.55362/IJE/2022/3781.

[fsn370328-bib-0006] Beta, T. , S. Nam , J. E. Dexter , and H. D. Sapirstein . 2005. “Phenolic Content and Antioxidant Activity of Pearled Wheat and Roller‐Milled Fractions.” Cereal Chemistry 82, no. 4: 390–393. 10.1094/CC-82-0390.

[fsn370328-bib-0007] Bhunia, R. K. , K. Sinha , R. Kaur , S. Kaur , and K. Chawla . 2023. “A Holistic View of the Genetic Factors Involved in Triggering Hydrolytic and Oxidative Rancidity of Rice Bran Lipids.” Food Reviews International 39, no. 1: 441–466. 10.1080/87559129.2021.1915328.

[fsn370328-bib-0008] Blüher, M. , and M. Stumvoll . 2020. “Diabetes and Obesity.” In Diabetes Complications, Comorbidities and Related Disorders, 1–49. Springer International Publishing.

[fsn370328-bib-0009] Demir, M. K. , N. Bilgiçli , S. Türker , and B. Demir . 2019. “Storage Properties of Wheat Germ Applied of Different Stabilization Processes.” Necmettin Erbakan University Journal of Science and Engineering 1, no. 2: 67–75.

[fsn370328-bib-0010] Demir, M. K. , and A. Elgün . 2013. “Stabilization of Whole Wheat Flour Branny Fractions With Special Emphasis on Internal and External Characteristic of Whole Wheat Bread.” Food Science and Technology Research 19, no. 2: 195–200. 10.3136/fstr.19.195.

[fsn370328-bib-0011] Demir, M. K. , and A. Elgün . 2014. “Comparison of Autoclave, Microwave, IR and UV‐C Stabilization of Whole Wheat Flour Branny Fractions Upon the Nutritional Properties of Whole Wheat Bread.” Journal of Food Science and Technology 51: 59–66. 10.1007/s13197-011-0475-0. 24426048 PMC3857423

[fsn370328-bib-0012] El‐Mahis, A. , M. H. Baky , and M. A. Farag . 2023. “How Does Rye Compare to Other Cereals? A Comprehensive Review of Its Potential Nutritional Value and Better Opportunities for Its Processing as a Food‐Based Cereal.” Food Reviews International 39, no. 7: 4288–4311. 10.1080/87559129.2021.2023817.

[fsn370328-bib-0013] Ertaş, N. 2015. “Effect of Wheat Bran Stabilization Methods on Nutritional and Physico‐Mechanical Characteristics of Cookies.” Journal of Food Quality 38, no. 3: 184–191. 10.1111/jfq.12130.

[fsn370328-bib-0014] Fărcaș, A. , G. Drețcanu , T. D. Pop , B. Enaru , S. Socaci , and Z. Diaconeasa . 2021. “Cereal Processing By‐Products as Rich Sources of Phenolic Compounds and Their Potential Bioactivities.” Nutrients 13, no. 11: 3934. 10.3390/nu13113934. 34836189 PMC8621182

[fsn370328-bib-0015] Francis, F. J. 1998. “Color Analysis.” In Food Analysis, edited by S. S. Nielsen , 599–612s. An Aspen Publishers.

[fsn370328-bib-0016] Goswami, M. , B. D. Sharma , S. K. Mendiratta , and V. Pathak . 2021. “Quality Improvement of Refined Wheat Flour Cookies With Incorporation of Functional Ingredients.” Journal of Food Processing and Preservation 45, no. 4: e14945. 10.1111/jfpp.14945.

[fsn370328-bib-0017] Gyamfi, M. A. , M. Yonamine , and Y. Aniya . 1999. “Free‐Radical Scavenging Action of Medicinal Herbs From Ghana: Thonningia Sanguinea on Experimentally‐Induced Liver Injuries.” General Pharmacology: The Vascular System 32, no. 6: 661–667. 10.1016/S0306-3623(98)00238-9. 10401991

[fsn370328-bib-0018] Hadidi, M. , S. R. Garcia , D. Ziogkas , D. J. McClements , and A. Moreno . 2024. “Cereal Bran Proteins: Recent Advances in Extraction, Properties, and Applications.” Critical Reviews in Food Science and Nutrition 64, no. 29: 10583–10607. 10.1080/10408398.2023.2226730. 37366171

[fsn370328-bib-0019] Haug, W. , and H. J. Lantzsch . 1983. “Sensitive Method for the Rapiddetermination of Phytate in Cereals and Cereal Products.” Journal of the Science of Food and Agriculture 34, no. 12: 1423–1426. 10.1002/jsfa.2740341217.

[fsn370328-bib-0020] Iraklı, M. , A. Lazaridou , and C. G. Biliaderis . 2020. “Comparative Evaluation of the Nutritional, Antinutritional, Functional, and Bioactivity Attributes of Rice Bran Stabilized by Different Heat Treatments.” Food 10, no. 1: 57. 10.3390/foods10010057. PMC7824238 33379306

[fsn370328-bib-0021] Jimenez‐Pulido, I. J. , R. Daniel , J. Perez , C. Martínez‐Villaluenga , D. De Luis , and A. B. Martín Diana . 2022. “Impact of Protein Content on the Antioxidants, Anti‐Inflammatory Properties and Glycemic Index of Wheat and Wheat Bran.” Food 11, no. 14: 2049. 10.3390/foods11142049. PMC9322734 35885294

[fsn370328-bib-0022] Kong, F. , L. Wang , H. Chen , and X. Zhao . 2021. “Improving Storage Property of Wheat Bran by Steam Explosion.” International Journal of Food Science and Technology 56, no. 1: 287–292. 10.1111/ijfs.14630.

[fsn370328-bib-0023] Kumar, S. , P. Seluriyal , S. Sharma , et al. 2023. “Functional and Nutritional Prospectives of Low‐Fat Cookies Fortified With Jamun Pulp, Jamun Seed, Mango Kernel Powder.” Applied Food Research 3, no. 2: 100340. 10.1016/j.afres.2023.100340.

[fsn370328-bib-0024] Leszczyńska, D. , A. Wirkijowska , A. Gasiński , D. Średnicka‐Tober , J. Trafiałek , and R. Kazimierczak . 2023. “Oat and Oat Processed Products—Technology, Composition, Nutritional Value, and Health.” Applied Sciences 13, no. 20: 11267. 10.3390/app132011267.

[fsn370328-bib-0025] Levent, H. , M. Koyuncu , N. Bilgicli , E. Adıgüzel , and M. Dedeoğlu . 2020. “Improvement of Chemical Properties of Noodle and Pasta Using Dephytinized Cereal Brans.” LWT 128: 109470. 10.1016/j.lwt.2020.109470.

[fsn370328-bib-0026] Li, Y. , Y. Zhang , L. Dong , et al. 2024. “Fermentation of *Lactobacillus fermentum* NB02 With Feruloyl Esterase Production Increases the Phenolic Compounds Content and Antioxidant Properties of Oat Bran.” Food Chemistry 437: 137834. 10.1016/j.foodchem.2023.137834. 37897817

[fsn370328-bib-0027] Makowska, A. , A. Polcyn , S. Chudy , and J. Michniewicz . 2015. “Application of Oat, Wheat and Rye Bran to Modify Nutritional Properties, Physical and Sensory Characteristics of Extruded Corn Snacks.” Acta Scientiarum Polonorum. Technologia Alimentaria 14, no. 4: 375–386. 10.17306/J.AFS.2015.4.37. 28068043

[fsn370328-bib-0028] Martín‐Diana, A. B. , M. J. García‐Casas , C. Martínez‐Villaluenga , J. Frías , E. Peñas , and D. Rico . 2021. “Wheat and Oat Brans as Sources of Polyphenol Compounds for Development of Antioxidant Nutraceutical Ingredients.” Food 10, no. 1: 115. 10.3390/foods10010115. PMC7828044 33430507

[fsn370328-bib-0029] Mathews, R. , and Y. Chu . 2020. “Global Review of Whole Grain Definitions and Health Claims.” Nutrition Reviews 78, no. Supplement_1: 98–106. 10.1093/nutrit/nuz055. 32728741

[fsn370328-bib-0030] Oghbaei, M. , and J. Prakash . 2016. “Effect of Primary Processing of Cereals and Legumes on Its Nutritional Quality: A Comprehensive Review.” Cogent Food & Agriculture 2, no. 1: 1136015. 10.1080/23311932.2015.1136015.

[fsn370328-bib-0031] Özkaya, H. , B. Özkaya , B. Duman , and S. Turksoy . 2017. “Effect of Dephytinization by Fermentation and Hydrothermal Autoclaving Treatments on the Antioxidant Activity, Dietary Fiber, and Phenolic Content of Oat Bran.” Journal of Agricultural and Food Chemistry 65, no. 28: 5713–5719. 10.1021/acs.jafc.7b01698. 28651042

[fsn370328-bib-0032] Ragaee, S. , K. Seetharaman , and E. S. M. Abdel‐Aal . 2014. “The Impact of Milling and Thermal Processing on Phenolic Compounds in Cereal Grains.” Critical Reviews in Food Science and Nutrition 54, no. 7: 837–849. 10.1080/10408398.2011.610906. 24499063

[fsn370328-bib-0033] Rico, D. , A. Villaverde , C. Martinez‐Villaluenga , et al. 2020. “Application of Autoclave Treatment for Development of a Natural Wheat Bran Antioxidant Ingredient.” Food 9, no. 6: 781. 10.3390/foods9060781. PMC7353647 32545426

[fsn370328-bib-0034] Saka, M. , B. Özkaya , and İ. Saka . 2021. “The Effect of Bread‐Making Methods on Functional and Quality Characteristics of Oat Bran Blended Bread.” International Journal of Gastronomy and Food Science 26: 100439. 10.1016/j.ijgfs.2021.100439.

[fsn370328-bib-0035] Saroj, R. , S. Kaur , M. A. Malik , V. Puranik , and D. Kaur . 2024. “Thermal Processing of Wheat Bran: Effect on the Bioactive Compounds and Dietary Fiber.” Bioactive Carbohydrates and Dietary Fibre 32: 100433. 10.1016/j.bcdf.2024.100433.

[fsn370328-bib-0036] Sözer, N. , L. Cicerelli , R. L. Heiniö , and K. Poutanen . 2014. “Effect of Wheat Bran Addition on In Vitro Starch Digestibility, Physico‐Mechanical and Sensory Properties of Biscuits.” Journal of Cereal Science 60, no. 1: 105–113. 10.1016/j.jcs.2014.01.022.

[fsn370328-bib-0037] Stephen, A. M. , M. M. J. Champ , S. J. Cloran , et al. 2017. “Dietary Fibre in Europe: Current State of Knowledge on Definitions, Sources, Recommendations, Intakes and Relationships to Health.” Nutrition Research Reviews 30, no. 2: 149–190. 10.1017/S095442241700004X. 28676135

[fsn370328-bib-0038] Subtain, M. , I. Pasha , F. Ahmad , et al. 2024. “Phytochemical Characterization and End Use Evaluation of Native and Fermented Cereal Brans.” Journal of Food Measurement and Characterization 18, no. 7: 5552–5563. 10.1007/s11694-024-02587-7.

[fsn370328-bib-0039] Sztupecki, W. , L. Rhazi , F. Depeint , and T. Aussenac . 2023. “Functional and Nutritional Characteristics of Natural or Modified Wheat Bran Non‐Starch Polysaccharides: A Literature Review.” Food 12, no. 14: 2693. 10.3390/foods12142693. PMC10379113 37509785

[fsn370328-bib-0040] Van Hung, P. 2016. “Phenolic Compounds of Cereals and Their Antioxidant Capacity.” Critical Reviews in Food Science and Nutrition 56, no. 1: 25–35. 10.1080/10408398.2012.708909. 25075608

[fsn370328-bib-0041] Wang, T. , F. He , and G. Chen . 2014. “Improving Bioaccessibility and Bioavailability of Phenolic Compounds in Cereal Grains Through Processing Technologies: A Concise Review.” Journal of Functional Foods 7: 101–111. 10.1016/j.jff.2014.01.033.

